# Supplementary material for: Iterative improvement in the automatic modular design of robot swarms
Source: PeerJ Comput Sci. 2020 Dec 7;6:e322. doi: 10.7717/peerj-cs.322 (PMC7924708; doi:10.7717/peerj-cs.322)
Supplement: Supplemental Information 3 [file peerj-cs-06-322-s003.zip › argos3/doc/api/standalone/a00485_source.html]

ARGoS: plugins/robots/foot-bot/simulator/qtopengl\_footbot.h Source File


- Main Page
- Related Pages
- Namespaces
- Classes
- Files

- File List
- File Members

# plugins/robots/foot-bot/simulator/qtopengl\_footbot.h

Go to the documentation of this file.

```
00001 
00007 #ifndef QTOPENGL_FOOTBOT_H
00008 #define QTOPENGL_FOOTBOT_H
00009 
00010 namespace argos {
00011    class CQTOpenGLFootBot;
00012    class CFootBotEntity;
00013 }
00014 
00015 #ifdef __APPLE__
00016 #include <gl.h>
00017 #else
00018 #include <GL/gl.h>
00019 #endif
00020 
00021 namespace argos {
00022 
00023    class CQTOpenGLFootBot {
00024 
00025    public:
00026 
00027       CQTOpenGLFootBot();
00028 
00029       virtual ~CQTOpenGLFootBot();
00030 
00031       virtual void Draw(CFootBotEntity& c_entity);
00032 
00033    protected:
00034 
00039       void MakeWheel();
00040 
00042       void SetWhitePlasticMaterial();
00044       void SetBlackTireMaterial();
00046       void SetCircuitBoardMaterial();
00048       void SetLEDMaterial(GLfloat f_red, GLfloat f_green, GLfloat f_blue);
00049 
00051       void RenderWheel();
00053       void RenderTrack();
00055       void RenderBase();
00057       void RenderGrippableSlice();
00059       void RenderGripperMechanics();
00061       void RenderGripperClaw();
00063       void RenderRAB();
00065       void RenderDistanceScannerSensor();
00067       void RenderDistanceScanner();
00069       void RenderIMX();
00071       void RenderBeacon();
00073       void RenderCamera();
00074 
00075    private:
00076 
00078       GLuint m_unLists;
00079 
00081       GLuint m_unBasicWheelList;
00082 
00084       GLuint m_unWheelList;
00086       GLuint m_unTrackList;
00088       GLuint m_unBaseList;
00090       GLuint m_unGrippableSliceList;
00092       GLuint m_unGripperMechanicsList;
00094       GLuint m_unGripperClawList;
00096       GLuint m_unRABList;
00098       GLuint m_unDistanceScannerSensorList;
00100       GLuint m_unDistanceScannerList;
00102       GLuint m_unIMXList;
00104       GLuint m_unBeaconList;
00106       GLuint m_unCameraList;
00107 
00110       GLuint m_unVertices;
00111 
00112       /* Angle gap between two leds */
00113       GLfloat m_fLEDAngleSlice;
00114 
00115    };
00116 
00117 }
00118 
00119 #endif
```

---

Generated on 10 Jul 2018 for ARGoS by 
 1.6.1
